# Acknowledgment to the Reviewers of *Journal of Imaging* in 2022

**DOI:** 10.3390/jimaging9020023

**Published:** 2023-01-20

**Authors:** Journal of Imaging Editorial Office

**Affiliations:** MDPI AG, St. Alban-Anlage 66, 4052 Basel, Switzerland

High-quality academic publishing is built on rigorous peer review. *Journal of Imaging* was able to uphold its high standards for published papers due to the outstanding efforts of our reviewers. Thanks to the efforts of our reviewers in 2022, the median time to first decision was 22 days and the median time from acceptance to publication was 4.8 days. Regardless of whether the articles they examined were ultimately published, the editors would like to express their appreciation and thank the following reviewers for the time and dedication that they have shown *Journal of Imaging*:
Abate, AndreaLeñero Bardallo, Juan AntonioAbbas, MuhammadLeon, RaquelAbboud, AliLeonardi, MarcoAbd El Aal, ShaabanLeuze, Christoph W. U.Abdulazeez, Adnan MohsinLeventić, HrvojeAbdullah, FaizanLin, Cheng ShianAbdullah, Johari YapLin, GuoqingAbedin, Kazi MonowarLiu, Chao J.Ackermann, JörgLiu, LiAdrien, JeromeLiu, LiqunAghzout, OtmanLiu, RongrongAhmed, WaelLiu, WeiweiAhn, Jae-KwangLiu, XiaoliAkagic, AmilaLiu, Yi KunAkao, YoshinoriLiu, YuhanAl-Ameen, ZohairLiu, YunAl-Amidie, MuthanaLizzi, FrancescaAlanafea, MohammadLoddo, AndreaAli, Md LiakatLozano, DemetrioAligholi, SaeedLu, TongtongAliotta, FrancescoLu, YuanrongAlmasri, HusseinLukosevicius, SauliusAlruwaili, MadallahMa, JackieAlshazly, HammamMa, XiaodongAlsufyani, NouraMaathuis, ClaraAlzubaidi, LaithMacairan, Jun-RayAmano, KinjiroMacek, WojciechAmer, HossamMachann, JürgenAmin, SikandarMacInnes, W. JosephAmiruzzaman, MdMadokoro, HirokazuAmyar, AmineMajnarić, IgorAnand, VijayakumarMakihara, YasushiAndres, FredericMakouei, FatemehAndujar, CarlosMarinov, PenchoAnejionu, ObinnaMario, PagnoniAngelidakis, VasileiosMarkötter, HenningAngelova, NadezhdaMarkov, KrassimirAnton, NicoletaMarques, BernardoAnyaiwe, Destiny E. O.Márquez, AndrésArandjelovic, OgnjenMarsili, StefaniaAravinda, C. V.Marta, DrążkowskaArmin, AliMartins, NunoArsalan, MuhammadMaruschak, PavloArteaga-Marrero, NataliaMaryada, Sai Kiran ReddyAslan, Muhammet FatihMasoumian, ArminAugusta, CarolynMatic, TomislavAvellino, IgnacioMaxa, JiříAziz, FurqanMay, DanielBachiller, Rocio EirosMazzinghi, AnnaBadža, Milica M.Megna, RosarioBae, Jang PyoMehta, Rupal I.Baek, Joong-HwanMendyk, AleksanderBaessato, FrancescaMichailidis, Panagiotis D.Baeßler, BettinaMichelucci, UmbertoBakator, MihaljMihai, IonBalaha, Hossam MagdyMiles, Brett A.Baliyan, VinitMillán-Millan, Pablo ManuelBallesteros, Dora M.Milosavljević, AleksandarBanterle, FrancescoMin, XiongkuoBarber, Paul RichardMirhashemi, ArashBarzekar, HoseinMirjalili, FereshtehBaspinar, EmreMitani, YoshihiroBauer, ZuriaMitianoudis, NikolaosBeilina, LarisaMohd-Isa, Wan-NoorshahidaBelaid, AbdelMoldovanu, SimonaBelarbi, Mohammed AminMoltisanti, DavideBelloulata, KamelMontisci, AugustoBenzaoui, AmirMoon, InkyuBerezin, AlexanderMorais, Pedro A.Berezsky, OlehMorgano, ManuelBernacki, BruceMoss, Alastair JamesBernecker, ClaudiaMouchère, HaroldBertelli, ElenaMoung, ErvinBerthier, MichelMousavi, ZohrehBerviller, YvesMustafa, Wan AzaniBi, GuoqiangNagaraju, RakeshBianconi, FrancescoNagy, Amr M.Blocker, Stephanie J.Nakamura, KensukeBlondet, CyrilleNapa, Sae-BaeBojarczak, PiotrNaqvi, Rizwan AliBonizzoni, LetiziaNarayanamurthy, Chittur SubramanianBonmati, EsterNaseri-Alavi, Seyed AhmadBopp, MiriamNawaz, MehmoodBosnar, DamirNeira-Tovar, LeticiaBotequim, DavidNg, Curtise Kin CheungBougourzi, FaresNg, Law YongBrad, RemusNguyen, Thai SonBraović, MajaNha, Pham VanBreuß, MichaelNi, KaiyuanBrioschi, MarcosNikaki, AlexandraBurn, FeliceNikolaidis, AthanasiosButaric, LaurenNiu, ChuangBuzzelli, MarcoNnamoko, NonsoCaccia, MicheleNombela, IvanCai, WenjieNoortman, Wyanne A.Caldeira, LilianaNordstrom, RobertCallahan-Flintoft, ChloeNovozhilova, NinaCallet, PatrickNowik, WitoldCapece, NicolaNuster, RobertCárdenas-Peña, DavidObukhov, Artem D.Cardone, AntonioOhsita, YuichiCarlinet, EdwinOishi, TakeshiCarrasco, MiguelOjah, JosephCattari, NadiaOkokpujie, Kennedy O.Cembranos, Jose A. R.Olatinwo, SegunCercenelli, LauraOliveira, Davi Ferreira DeChamchong, RapeepornOmer, Osama A.Chang, Tse-ShaoOn, Sung-WoonChang, YiOneață, DanChassain, CarineOostveen, JobChauhan, RituOprisescu, SerbanChelli, AliOsipov, Sergei PavlovichChen, Chao-PingÖztürk, ŞabanChen, ChongPacanowski, RomainChen, HaoPaek, Sung WookChen, Jian-HuaPalaiahnakote, ShivakumaraChen, Kuang-HsuanPalmieri, LucaChen, Ting-liPalnitkar, HarishCherepennikov, YuPan, ZhangChertovskih, RomanPandey, Om JeeChiverton, JohnPapadopoulos, TheofilosCho, HyeonjoongPastor, MoisésChoi, KiHongPaszkowski, RobertChoi, WookjinPatel, SamirChow, CaseyPatwardhan, Amol S.Chrcanovic, BrunoPaul, Joseph SureshChuang, Yung-ChungPawar, AishwaryaChuirazzi, WilliamPérez-Juste Abascal, Juan FelipeChung, Ki-SeokPero, ChiaraChung, KyungyongPerrotta, AndréCiecholewski, MarcinPescaru, DanCimarelli, ClaudioPesznyák, CsillaCiocca, GianluigiPeter, DineshCobus, LauraPeterlik, IgorColella, AbnerPetrellis, NikosComelli, AlbertPetro, Ana BelenComes, RaduPham, Cong-KhaComis, Alessio DaniloPhan, TinCondorelli, FrancescaPhilippe, CathyConstantoudis, VasiliosPhyo, Cho NilarContaldo, MariaPiccolomini, Elena LoliCorchs, SilviaPintus, RuggeroCorsonello, PasqualePiovarči, MichalCortea, Ioana MariaPitas, Charalampos N.Cöster, Maria C.Plut, DomenDabagh, MahsaPodurets, KonstantinDahmane, MohamedPoggio, Anna DelDamaševičius, RobertasPonce Gallegos, Julio CésarDanilevicz, Monica F.Positano, VincenzoDauvergne, DenisPouliakis, AbrahamDawwd, ShefaPovedano, Juan ManuelDe Araujo, Sidnei AlvesPowroznik, PawelDe Bernardi, ElisabettaPrakasa, EsaDe Frond, HannahPreda, Silviu DanielDe La Fraga, Luis G.Přibil, JiříDe Oliveira Teixeira, LucasPrieto, Andrés J.Decaestecker, ChristinePuppe, FrankDeglint, JasonQi, GuanqiuDel Pizzo, SilvioQian, YuleiDeng, KeRadil, RomanDesai, KevinRai, RobbaDesjardins, AdrienRaiyani, KashyapDewi, ChristineRakhmangulov, AleksandrDi Nardo, DarioRamanathan, ManojDi Trapani, VittorioRan, YupingDikaios, NikolaosRanaldi, LeonardoDileo, GiuliaRangaiah, PramodDobo-Nagy, CsabaRastad, HadithDobrea, Monica ClaudiaRavi, Renjith V.Dobrescu, GianinaReda, RodolfoDološ, KlaraReibman, AmyDong, QiuleiRenero-Carrillo, Francisco-J.Doumit, Jean A.Rensink, RonaldDumusc, AlexandreRespondek-Liberska, MariaDzierżak, RóżaRhanoui, MaryemEbenhan, ThomasRhee, Kang HyeonEck, UlrichRicci, SerenaEdwards, EddieRinaldi, Antonio MariaEgger, JanRisojevic, VladimirEl Alami, HassanRizuana, Iqbal Hussain El Mallahi, MostafaRodin, Vladislav GennadievichElbouz, MarwaRodríguez Valido, Manuel JesúsEleswarapu, Ananth S.Rogers, Thomas BashfordElezi, IsmailRohling, RobertElfer, KatherineRomán Gallego, Jesús ÁngelElmi-Terander, AdrianRössl, ChristianElshennawy, NadaRubins, UldisElwahab, Sami AbdRudan, JohnErappa, Shobha M.Rundo, LeonardoEresen, AydinRyu, Mun-HoErskine, Samuel KofiRyumin, DmitryEsfandiari, HoomanSaabni, RaidFadaei, SadeghSaadane, RachidFang, YuchunSabeenian, R. S.Farago, PaulSager, PascalFechteler, PhilippSahu, Aditya KumarFei, WenbinSakellaropoulou, AntigoniFeng, RuitaoSaleh, SadeqFenster, AaronSaligheh Rad, HamidFerrag, Mohamed AmineSalmon, Morgan D.Ferreira, MariaSandu, Irina Crina AncaFerreira, NunoSantoro, AdolfoFesta, GiuliaSantos, Max Mauro DiasFeuchtner, Gudrun M.Saraiva, Miguel MascarenhasFilip, JiriSarvia, FilippoFillingham, PatrickSaswata, MukherjeeFischer, JannisSavino, PasqualeFishman, DmytroSayyouri, MhamedFitton, IsabelleScapicchio, CamillaFletcher, AlexanderSchade, Gunnar W.Frattolillo, FrancoSchawkat, KhoschyFriebe, MichaelSchena, StefanoFrouin, VincentSchillinger, BurkhardFuh, Chiou-ShannSchiopu, IonutGaboury, SébastienSchoenberg, MarkusGallego, Óscar S.Scolavino, SalvatoreGalli, MassimoSeal, AyanGao, KunŠegota, Sandi BaressiGao, LinSeo, Byung-KukGao, PanShah, ChiranjibiGao, WeiSharma, HemantGao, WeidongSharma, NehaGarcía-Floriano, AndrésShchepetkina, AlinaGardašević-Filipović, MilankaShemer, AmirGargano, MarcoShen, Tsu-WangGeorgieva-Tsaneva, GalyaShen, XizhongGeradts, ZenoShendrik, RomanGermann, JürgenShi, HengcanGiannelli, MarcoShin, Byeong-SeokGigilashvili, DavitShin, SeungwooGijsenij, ArjanShirazi, Amin ZadehGiuliano, AlessiaSi, WeixinGlinz, JonathanSiegele, DietmarGłowacki, PrzemysławSik-Lanyi, CeciliaGodinho, Daniela M.Simone, GabrieleGordienko, Yuri G.Singh, SimrandeepsGötz, MichaelSinitsyn, Valentin Govil, HimanshuSk, Md ObaidullahGronau, NuritSmits, Robert G.Grosse, KathrinSoltaninejad, MohammadrezaGruschwitz, PhilippSong, BinGuo, YanhuiSopegno, AlessandroGuo, ZheSorantin, ErichGupta, DeepaSousa, AntónioGustschin, AlexSpagnolo, FannyHabijan, MarijaSpehar, BrankaHafeez, AzeemStanzione, ArnaldoHaim, HarelStaroletov, SergeyHaist, TobiasStateczny, Andrzej EugeniuszHajari, NasimStolarska, MagdalenaHajj, BassamStone, Nelson N.Hamilton, DaleStrigari, LidiaHamza, RafikSun, HongbinHamzah, Rostam AffendiSun, JiguangHanbay, KazimSun, XinHansen, Søren BaarsgaardSzava, JanosHaouchine, NazimSzemenyei, MártonHe, HongliangTagawa, NorioHe, QinghuaTaleby Ahvanooey, MiladHektor, JohanTamada, DaikiHelbig, MarkoTan, Phan XuanHelm, Patrick A.Tan, ShanshanHelmholz, PetraTang, FangfangHigson, EdwardTang, Yuk MingHochuli, Andre GustavoTango, FabioHodgson, AlanTao, YangHoegger, MarkTariq, AmaraHofer, EdithTaylor, RebeccaHofstad, Erlend FagertunTemerinac-Ott, MajaHolte, Michael BoelstoftThepade, SudeepHomayounvala, ElahehThielemans, KrisHong, RunyuThieu, ThanhHorvat, MarkoThomas, Hannah M.Hossain, Md AlamgirTian, JingHosseini, SaeidTokdemir, GulHotait, HassaneTörő, OlivérHsu, Quang-CherngTorres, IreneHu, Houchun HarryTran, Song ToanHu, YiTranouez, PierrickHuang, WeiminTreimer, WolfgangHughes, PaulTsai, MinjenHung, Chih-ChengTsotsos, John K.Hunter, DavidTsuneki, MasayukiHusár, JozefTudisco, ErikaIancu, VioletaTutwiler, RichardIkuesan, RichardTzortzakakis, AntoniosIlie, ConstantinÜncü, YiğitImran, Ali ShariqUnlu, SerkanImran, AzharUrien, HeleneInoue, KoheiValtierra-Rodriguez, MartinInoue, NaotoVanKoevering, Kyle KeithIntzes, IoannisVarga, DomonkosInzerillo, LauraVelev, Dimiter GeorgievIon, MarianVerger, AntoineIslam, MohammedVerlekar, TanmayIwahori, YujiViana, ThiagoIzzetti, RossanaViermetz, ManuelJabreel, MohammedVilagosh, ZoltanJamaludin, ShahrizanVilor-Tejedor, NataliaJamil, SonainVitulano, DomenicoJanowski, LucjanVolosencu, ConstantinJardin, AxelVora, KeyurJegelevičius, DariusVozel, DomenJerković, IvanWahaia, FaustinoJi, WeiWakabayashi, TaigaJian, MuweiWang, DezhiJoyce, Daniel S.Wang, GuoqiangJung, Ki-HyunWang, LeiKabra, SaurabhWang, LuluKakavas, SotiriosWang, XingyuKalaidzidis, YannisWei, JiaKalmoun, El MostafaWen, HanKang, Bong JooWen, SenfarKang, KyeongPilWitczak, PiotrKansal, PriyaWójcik, DariuszKara, İlkerWolfe, JeremyKarras, Dimitrios A.Wróblewska, AnnaKatic, MarkoWu, BingKatina, StanislavXie, ShengkunKawa, JacekXu, ZhengKawabata, NorifumiYaacob, Wan Fairos WanKemper, BjörnYakovyna, VitaliyKerekes, JohnYang, FanKesidis, Anastasios L.Yang, Fu-LiangKhalesi, BanafshehYang, JingKhalil, Magdy M.Yang, LiuqingKhamukhin, AlexanderYang, PengpengKhatami, HamidrezaYao, JingKhaykovich, BorisYe, TongKhishe, MohammadYennapureddy, Manoj KumarKim, Hyung-TaeYoshinaga, TetsuyaKim, Jong-ChanYou, ShingchernKim, JongwooYousuf, Mohammad AbuKim, Sang-WoonYu, ZekuanKirsten, Toralf Yu, ZidanKishta, WaleedYüzügüllü, OnurKlimaszewski, KrzysztofZabler, SimonKojdecki, MarekZambrano-Martinez, Jorge LuisKokoulin, Andrey N.Zandt, Rene In ’tKönig, AndreasZanza, AlessioKotova, SvetlanaZara, FrancescaKotowski, KrzysztofZenati, NadiaKozawa, YuichiZhang, FanglueKoziri, MariaZhang, FengqiaoKozlov, KonstantinZhang, GuangjuKrenc, KsaweryZhang, HanqingKröger, Jan RobertZhang, Jixian Krzeszowski, TomaszZhang, XiaohuiKubera, ElżbietaZhang, YanliKuriqi, AlbanZhao, TianKwa, KatherineZhao, ZuominKwon, Soon-kakZheng, ZeshiLa Cascia, MarcoZhong, LimingLajevardipour, AlirezaZhou, HaoyinLaManna, JacobZhou, ShijieLe, Nguyen Quoc KhanhZhou, ShoujunLe, Thi XiuZhou, TaoLeão, CelinaZhou, TianfeiLee, DeokwooZhou, YangLee, YounghoZhou, Yu-PengLeeuwen, Tristan VanZhu, WenliangLegland, DavidZlatev, ZlatinLei, YaohuZoric, BrunoLeich, AndreasZsedrovits, Tamas


